# Schizophrenia: Complement Cleaning or Killing

**DOI:** 10.3390/genes12020259

**Published:** 2021-02-11

**Authors:** Jirrine T.T. Hogenaar, Hans van Bokhoven

**Affiliations:** 1 Donders Institute for Brain, Cognition and Behaviour, Radboud university medical center, P.O. Box 9101, 6500 HB Nijmegen, The Netherlands; j.hogenaar@student.ru.nl; 2 Department of Human Genetics, Radboud university medical center, P.O. Box 9101, 6500 HB Nijmegen, The Netherlands

**Keywords:** schizophrenia, complement system, synaptic pruning

## Abstract

Schizophrenia is a psychiatric disorder with a typical onset occurring during adolescence or young adulthood. The heterogeneity of the disorder complicates our understanding of the pathophysiology. Reduced cortical synaptic densities are commonly observed in schizophrenia and suggest a role for excessive synaptic elimination. A major pathway hypothesised to eliminate synapses during postnatal development is the complement system. This review provides an overview of genetic and functional evidence found for the individual players of the classical complement pathway. In addition, the consequences of the absence of complement proteins, in the form of complement protein deficiencies in humans, are taken into consideration. The collective data provide strong evidence for excessive pruning by the classical complement pathway, contributing to cognitive impairment in schizophrenia. In future studies, it will be important to assess the magnitude of the contribution of complement overactivity to the occurrence and prevalence of phenotypic features in schizophrenia. In addition, more insight is required for the exact mechanisms by which the complement system causes excessive pruning, such as the suggested involvement of microglial engulfment and degradation of synapses. Ultimately, this knowledge is a prerequisite for the development of therapeutic interventions for selective groups of schizophrenia patients.

## 1. Introduction

Schizophrenia is a psychiatric disorder with a prevalence of about 1% and an estimated heritability of around 80% [1]. The key clinical features of schizophrenia are negative symptoms (social withdrawal), positive symptoms (hallucinations and delusions), and cognitive impairment. Understanding the mechanisms underlying this developmental disorder is of importance for the innovation of effective treatments to alleviate patients’ impairments. A striking characteristic of schizophrenia is the initiation of symptoms during adolescence and young adulthood. Although schizophrenia has been called a developmental disorder already in 1873 by Thomas Clouston, who used the term adolescent insanity [2,3], the pathophysiology of this disorder has yet to be fully deciphered. 

A well-known system thought to be hyper-responsive in schizophrenia is the dopamine system. Antipsychotic drugs target dopamine D2 receptors and aggravate psychosis in schizophrenia [4]. However, another hypothesis that fits the age of onset was formulated by Feinberg [5]. Schizophrenia onset during adolescence could be the result of impaired maturation during neurodevelopment. The first hinting towards brain physiology changes during adolescence emerged from Feinberg’s sleep studies, demonstrating electroencephalogram (EEG) changes during early infancy and adolescence [6]. Feinberg’s point of view was that the vulnerability of adolescents might well be the result of a dramatic brain re-organisation that takes place during this period [5]. In addition, it was found that during adolescence the cortical synaptic density reduced disproportionally [5,7]. This reduction in synapses can result from impaired synaptogenesis or be the consequence of overactive synapse elimination by faulty pruning mechanisms. 

Although non-genetic factors can play a role in the aetiology of schizophrenia [8], the aggregation of schizophrenia in families are a strong indication of a genetic contribution [9]. The majority of schizophrenia cases are thought to have a multifactorial aetiology, but rare cases have been identified in which single genetic or genomic variants have a major contribution to the occurrence of schizophrenia. The best known example is the 22q11.2 deletion syndrome (22q11DS), which is associated with an estimated 25-fold increased risk for schizophrenia and which may account for 0.5–1% of all schizophrenia cases [10]. In addition, rare and de novo intragenic variants have been identified in a low proportion of schizophrenia cases, examples including *SETD1A* [11] and *Disrupted-In-Schizophrenia 1* (*DISC1*) [12]. Yet, the majority of cases are thought to be attributed by combinations of multiple rare and common variants. Genome-wide association studies (GWAS) demonstrated a genetic association with schizophrenia for multiple loci [13]. As recently addressed by Woo et al. [14], genetic studies have identified several complement-related risk loci for schizophrenia. The strongest genetic relationship for schizophrenia is with genetic markers located at the major histocompatibility complex (MHC) locus [15], which contains four genes that are related to the complement system (complement factor *B*, *C2*, *C4A*, and *C4B*) [14]. To clarify the association of schizophrenia to the MHC locus, Sekar et al. focused on human C4 [15]. Combining their evidence on the association of schizophrenia to *C4*, or *C4A* more specifically, with previous evidence on C1q-mediated postnatal synaptic pruning [16], provided a strong indication for the involvement of the classical pathway of the complement system in schizophrenia. 

This review addresses the question whether hyperactivity of the classical pathway of the complement system contributes to the pathophysiology of schizophrenia through excessive synaptic pruning during postnatal development. We provide an overview of the evidence found for complement-mediated synaptic pruning during postnatal development and discuss the specificity of the classical pathway’s role in the pathophysiology of schizophrenia. 

## 2. The Three Pathways of the Complement System

**Figure 1:**
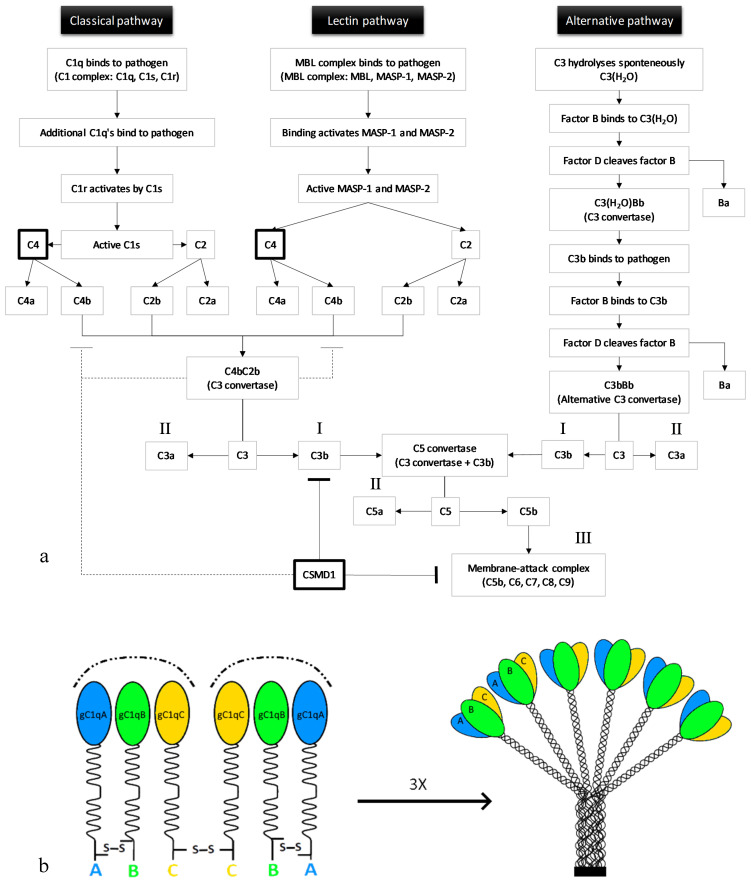
(**a**) The three pathways of the complement system. All three pathways can lead to C3b opsonisation of a target cell through the formation of a C3 convertase (C4bC2b or C3bBb). Subsequently, the C5 convertase can be formed by binding of C3b to a C3 convertase. C5b, together with C6, C7, C8, and a C9-polymer, generates the membrane attack complex (MAC). A tight regulation of the complement activation is needed to protect healthy host cells from phagocytosis and cell lysis. (**b**) Visualisation of the complement protein C1q composition. C1q consists of three peptide chains (A, B, and C). Disulfide bridges (S-S) connect the N-terminals of A- and B-chains and two C-chains. The C-terminals are globular domains (gC1qA, gC1qB, and gC1qC) that recognise immune targets. Adapted from Frachet et al. (2015) [19].

The complement system is formed by a group of plasma proteins that interact with each other to remove pathogens or unwanted cells. Complement proteins can be either soluble or membrane-associated [17]. Upon inflammation, proteases that are part of the system are activated through proteolytic cleavage. Then, these proteases react in a cascade, one protease can activate another through cleavage, thereby amplifying the signal. There are three complement pathways: The classical, the lectin, and the alternative pathway. Each pathway leads to C3b opsonisation of pathogens (recruits phagocytes) (Figure 1a, I), anaphylatoxin production (promotes inflammation) (Figure 1a, II), and membrane-attack complex (MAC) formation (leads to cell lysis) (Figure 1a, III) [18]. 

The first component of the classical pathway is C1q. C1q can either bind to antibodies interacting with antigens or to the pathogen’s surface directly [20]. Together with two C1r and two C1s zymogens (enzyme precursors), C1q forms the C1 complex [21]. Multiple C1qs bound to one pathogen cause a conformational change leading to the activation of C1s by C1r. C1s then cleaves C4, generating C4a and C4b. C4b, bound to the target’s surface, interacts with C2, thereby enabling C1s to also cleave C2 (resulting in C2a and C2b). The large subunits, C4b and C2b, together form a C3 convertase [20]. 

The first molecule of the lectin pathway, mannan-binding lectin (MBL), is very similar to C1q. MBL is also in a complex with two zymogens, MBL-associated serine proteases 1 and 2 (MASP-1 and MASP-2), called the MBL complex. Binding of the MBL complex to a pathogen’s surface activates the zymogens after which these cleave C4 and C2, again producing C4b and C2b that together form a C3 convertase. Both C4b and C3b are rapidly inactivated when they are not bound to the pathogen’s surface, protecting host cells from opsonisation [20]. 

The alternative pathway is not initiated by the binding of a pathogen. Instead, C3 hydrolyses spontaneously. The conformational change upon hydrolysation enables the binding of plasma protein factor B to C3(H_2_O). This interaction allows the cleavage of factor B by factor D, producing Ba and Bb. C3(H_2_O) and Bb together form the C3(H_2_O)Bb complex, which is an unstable C3 convertase. A fraction of the C3b which is produced by this convertase binds covalently to the host cells or pathogens. This C3b can bind factor B, as did C3(H_2_O), again allowing factor D to cleave factor B. The alternative pathway C3 convertase is formed by C3bBb. The alternative pathway can function as an amplification loop for the two other pathways [20].

Thus, each of the pathways produces a C3 convertase. The cleavage of C3 results in abundant covalent binding of C3b proteins to the surface of a target cell. C3b molecules opsonise the target cell, creating a target for C3b-receptor-carrying phagocytes [20]. In addition to the opsonisation of target cells, C3b production also initiates the next event of the complement system by binding to a C3 convertase, which leads to the formation of a C5 convertase. C5 interacts with C3b in the C5 convertase complex, after which either C3b or Bb cleaves C5 into C5a and C5b [20]. The production of C5b initiates the assembly of the MAC (C5bC6C7C8C9), that disrupts cellular homeostasis upon permeabilisation of the membrane. Furthermore, degrading enzymes will be able to enter the cell, ultimately leading to cell lysis. Regulation of the complement system is achieved through prevention of formation of C3 convertase or MAC, protecting host cells from compliment activation [20]. 

## 3. Synaptic Pruning during Postnatal Development

Evidence has implicated the complement system in the pathophysiology of schizophrenia through the elimination of synapses during postnatal development of the central nervous system (CNS). Synaptic pruning is the process of removing redundant synapses. Postnatal synaptic pruning was first demonstrated in monkeys: In the rhesus monkey, it was shown that the number of synapses, throughout the cortex, increases postnatally and then decreases during adolescence before the amount of synapses stabilises in early adulthood [22]. These observations were confirmed in humans, showing a prolonged decline especially in prefrontal, temporal, and parietal regions [23,24]. Importantly, it has been shown that developmental remodeling in humans might take even longer than in non-human primates, continuing throughout the third decade of life [25]. In addition, individuals at clinical risk for developing psychosis showed steeper rates of reduction in cortical thickness (grey matter) compared to individuals who did not develop psychosis [26]. This was seen for the right superior frontal, middle frontal, and medial orbitofrontal regions. The amount of grey matter loss in prefrontal, hippocampal, and thalamic areas can be explained by the dendritic loss, of which the integrity is dependent on the integrity of synapses [27]. 

Correct synaptic pruning is essential for the maturation of neural networks. The first important steps towards elucidating molecular mechanisms of synaptic pruning were taken by studying the neuromuscular junction (NMJ) in the peripheral nervous system [28]. These studies provided evidence for an activity-dependent mechanism controlling synapse elimination [29,30]. Axonal remains at the NMJ were engulfed by Schwann cells, indicating a role for glial cells in axon removal at the NMJ [31]. Using the mouse retinogeniculate (RGC) system, Stevens et al. identified a role for astrocytes and proteins of the complement system as mediators of synapse elimination [16]. 

In the next chapters, we will focus in more detail on the potential role of the individual players of the classical pathway of the complement system in synaptic pruning and the consequences of complement deficiencies. 

## 4. Complement Protein C1q: The Initiating Protein of the Classical Pathway 

The C1q subunit of the C1 complex consists of three highly homologous proteins: C1qA, C1qB, and C1qC [32]. C1q is formed by 18 polypeptide chains in total: Six A-chains, six B-chains, and six C-chains [19] (Figure 1b). C1q as a contributing factor in synaptic pruning was first identified by Stevens et al. [16]. Gene profiling of RGCs that were exposed to astrocytes showed the upregulation of all three C1q chains (A, B, and C) [16]. *C1q* messenger RNA (mRNA) levels were significantly reduced at postnatal day 30 (P30) compared to P5 and P10 RGCs, suggesting that *C1q* is not expressed by adult RGCs. Immunofluorescence indicated that C1q is more often colocalised with small, immature synapses identified by either presynaptic SV2 or postsynaptic density protein-95 (PSD95) than with mature synapses in the developing dorsal lateral geniculate nucleus (dLGN). The localisation of C1q to developing synapses together with the knowledge that C1q in the classical complement cascade is able to remove cells, suggested that C1q functions in synapse removal in the developing RGC system. In support of this, the dLGN of *C1q* knockout mice showed a significant overlap between contralateral and ipsilateral RGC projections, a segregation deficit which is indicative of a pruning deficiency. Patch-clamp recordings showed that dLGN neurons received an increased number of inputs in the C1q knockout mice compared to the wildtype mice, strengthening the observation that C1q deficiency results in a segregation deficit, suggestive of reduced synaptic pruning in the RGC system [16]. Pruning failure in the absence of C1q was further investigated by Chu et al. [33]. They showed increased axonal lengths and smaller inter-bouton (bouton = presynaptic axon terminal) distances in layer V pyramidal cells in *C1q* knockout mice. The increased axonal bouton density in these mutants could be the result of pruning deficits in the cortex, confirming an important role for C1q in synaptic pruning [33]. 

In a follow-up study on the pioneering work from Stevens et al. [16], it was shown that transforming growth factor beta (TGF-β) signaling stimulates *C1q* expression in microglia, thus controlling the elimination of synapses in a complement-dependent manner [34]. A more recent study used C1q-tagged isolated synaptic terminals (synaptosomes) to characterise their molecular composition. Mass spectrometry of untagged and C1q-tagged synapses revealed 18 proteins with different abundances between the two groups of synaptosomes. The bioinformatics analysis of downstream pathways of these proteins identified apoptosis and synaptic plasticity as possible converging downstream pathways. These expression profiles suggest the occurrence of local biochemical changes in synaptic compartments without neuronal death, also known as synaptic apoptosis or synaptosis colocalization of C1q and apoptotic markers such as caspase-3, the main effector molecule of apoptosis, was confirmed in the human cerebral cortex and in barrel cortices of mice in which pruning was induced by the removal of whiskers [35]. In addition, Annexin V and C1q appeared to compete for phosphatidylserine binding, as the presence of Annexin V reduced the number of C1q-positive synaptosomes, suggesting that C1q binds to phosphatidylserine exposed on synapses after initiation of apoptosis [35] as exposed phosphatidylserine forms a characteristic of apoptotic cells.

These data suggest that C1q does not only function in the host defence as part of the innate immune system, but also in the postnatal development of neuronal circuits in the CNS. This notion is shared by Sager et al. [36] who observed a postnatal increase in classical complement factors in the human dorsolateral prefrontal cortex congruent with their predisposing role in schizophrenia [36]. It will be important to determine through which mechanisms C1q affects synaptic pruning and whether C1q is a marker for schizophrenia.

## 5. Complement Protein C4

**Figure 2:**
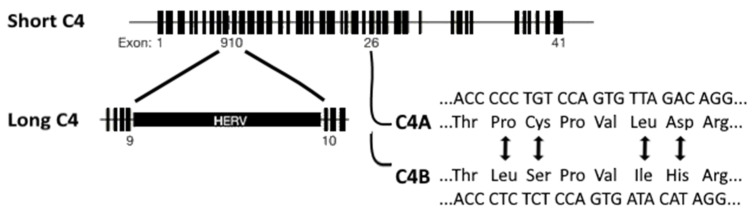
Isotypes of human C4 gene located on chromosome 6. C4A and C4B can be distinguished by amino acid differences. Long genomic forms of either C4A or C4B include an HERV insertion in intron 9. Image adapted from Sekar et al. [15].

As mentioned in the introduction, Sekar et al. [15] investigated the association of schizophrenia to the MHC locus by focusing on human C4, which is also located at this locus on chromosome 6p21.3 [15]. The two isotypes of human C4 are C4A and C4B. Of these isotypes, long and short genomic forms exist: C4AL (long), AS (short), BL, and BS (Figure 2). The long forms contain an intronic human endogenous retroviral (HERV) insertion that does not change the C4 protein sequence. The *C4A* and *C4B* loci vary in copy number and HERV-status. The four most common C4 haplotypes, identified through the analysis of father-mother-offspring trios, are AL-BL, AL-BS, AL-AL, and BS. A post mortem human adult brain sample investigation revealed that increased copy numbers increased *C4* expression proportionally. Moreover, mRNA levels of *C4A* were 2–3 times higher than those of *C4B* [15].

Using integrated haplotypes of MHC single nucleotide polymorphisms (SNPs) and *C4* alleles for an association assessment, the strongest association was seen for a large set of SNPs at the distal end of the extended MHC region. The next strongest association peak was found at C4. SNPs that correlated more strongly with the genetically predicted *C4A* expression, were more strongly associated with schizophrenia. Furthermore, individual *C4* alleles also showed schizophrenia risk, independent of the MHC haplotype at which they were located. These findings suggest that *C4A* expression is elevated in schizophrenia patients, which was confirmed by measuring the *C4* expression in human brain tissue. The *C4A* expression was 1.3-fold greater in individuals with schizophrenia compared to individuals without schizophrenia.

Next, the C4 distribution in the human brain was determined using vesicular glutamate transporter 1/2 (presynaptic marker), PSD95, and NeuN, a neuronal marker present in the nuclei and perinuclear cytoplasm of neurons [37], which showed C4 localisation at neurons and synapses in the human brain [15]. In line with the C1q results [16], *C4* RNA was expressed in the LGN and in RGCs which were purified from the retina of mice. Additionally, C4 deficiency reduced immunostaining of its effector C3 in the dLGN. Finally, C4 deficiency also resulted in a segregation deficit: There was a greater overlap between ipsilateral and contralateral RGC inputs in the dLGN of *C4*^−/−^ mice. Heterozygous *C4*^+/−^ mice showed an intermediate phenotype for the level of input overlap [15]. These data reveal a role for C4 in schizophrenia and support the hypothesis of complement-mediated pruning being involved in schizophrenia pathology.

The discoveries of Sekar et al. prompted the determination of *C4A* mRNA expression in patients with schizophrenia, bipolar disorder with psychosis, and controls [38]. A positive correlation was found between *C4A* expression and the positive factor scores from the positive and negative syndrome scale (PANSS). In addition, regression modelling showed that *C4A* primarily contributed to the severity of delusions. The second strongest association was seen for hallucinations. The authors suggested that an elevated *C4A* expression might contribute to the development of psychosis through excessive pruning [38]. In addition, an increased *C4A* RNA expression correlated with poorer episodic memory performance and reduced cortical activity in the middle temporal gyrus upon a visual processing task [39]. This suggests the complement system implication in cognitive impairment seen in schizophrenia. 

A follow-up on the study by Sekar et al. [15] investigated the relationship of *C4* gene copy numbers with neuropil contraction and expansion in the human cortex [40]. For this, two independent cohorts of schizophrenia patients (young adult-onset and adolescent-onset schizophrenia) and age-matched healthy controls were included [40]. Prasad et al. [40] examined neuropil since this is better achievable in humans than measuring synapses. Neuropil was measured through the amount of available membrane phospholipid (MPL) precursors and catabolites. Neuropil growth is associated with the MPL synthesis, whereas neuropil breakdown increases the level of catabolites. Changes in axonal endings, dendritic branches, and synapses are thought to be the main contributors to neuronal membrane expansion and contraction. However, Prasad’s hypothesis that increased *C4A* repeats would increase the amount of available MPL precursors and decrease the level of catabolites in the cerebellum and frontal, cingulate, parietal, and orbitofrontal cortex was not fully supported. They only observed neuropil contraction in the prefrontal and parietal regions of adult-onset patients and in the prefrontal and thalamic regions of adolescent-onset patients [40]. 

A different approach to identify differential complement profiles is to compare the plasma protein composition of psychiatric patients with healthy controls before clinical onset. A proteomic study was performed on plasma samples of children [41]. Decreased C4b-binding protein alpha chain (C4BPA) and C4b-binding protein beta chain (C4BPB) levels were found in children preceding the onset of psychotic disorder [41]. C4BPA and C4BPB together form the C4b-binding protein. This protein inhibits the classical and lectin pathway through C4b-binding, preventing C3 convertase formation [42]. The pathway analysis identified the “Complement and Coagulation Cascade” as a top hit among 60 significantly different expressed proteins between psychotic patients and controls [41]. 

In short, *C4A* expression was found to be elevated in schizophrenia, C4 was localised at synapses and neurons in the human brain, C4 deficiency produced a segregation deficit in the mouse RGC system (as did C1q), *C4A* expression was positively associated with schizophrenia symptoms (delusions and reduced memory performance), decreased C4b inhibiting protein levels were found in children preceding a psychotic disorder, and increased *C4A* repeats were associated with neuropil contraction in multiple brain regions of schizophrenic patients. Together these data support a role for C4, probably via the complement system, in the pathophysiology of schizophrenia. 

## 6. Complement Protein C2

In contrast to C1 and C4, C2 is not well investigated in the context of schizophrenia and two studies involving C2 in schizophrenic patients had contradictory outcomes. The first study found a significantly increased activity for C2 (and C1, C1q, C3, C4) in schizophrenia patients compared to the healthy controls [43], whereas a second study found the C2 activity to be significantly lower in patients compared to the controls [44]. The majority of individuals with C2 deficiency (>60%) do not suffer from the apparent disease. Most of the individuals that are affected develop a systemic autoimmune disease. It is possible that Factor B, the alternative pathway homologue of C2, is able to compensate for a lack of C2 in most individuals [45]. The effects of complement deficiency are further addressed in the last chapter: Schizophrenia and autoimmune disorders. 

## 7. Complement Protein C3

Following the identification of C1q as a potential player in synaptic pruning in the CNS [16], further investigations addressing the question whether C1q acts through the complement system provided evidence for C1q-C3 mediated pruning in the developing CNS. A follow-up study linked C3 mediated pruning to presynaptic engulfment by microglia [46]. The hypothesis that microglia prune through the C3 receptor (CR3) was strengthened by the use of minocycline, which inhibits the microglial activity [47], as this significantly reduced the RGC input engulfment [46]. Together these data propose that the RGC input engulfment is mediated by both neural activity and the complement system [16,46], which is a plausible underlying mechanism of synaptic pruning in developing neural circuits. However, *CR3* and *C3* knockout mice showed reduced engulfment, and not absence of engulfment. This indicates that synaptic engulfment is not CR3 or C3 dependent, suggesting that other mechanisms are involved in synaptic engulfment, as well. 

A role for C3 has been put forward as a factor for the cognitive phenotype in individuals with schizophrenia with learning and memory deficits as a core symptom. Memory is a heritable trait in which long-term potentiation (LTP) is a cellular mechanism [48]. During LTP, increased *C3* and *MHC* class *I* and *II* expression was identified in the hippocampus of live rats [49]. Investigation of the genetic association between the activation of C3 in schizophrenia revealed the strongest associations with the CUB and Sushi multiple domains 1 and 2 (*CSMD1/2*) genes [48]. The strong association between the *CSMD1/2* and schizophrenia was confirmed in the large unbiassed GWAS by the Psychiatric Genomics Consortium [50]. Moreover, a genetic variant in *CSMD1* (rs2740931) was found to be study-wide and significantly associated with an episodic memory [51]. 

A repeated pattern of complement control protein (CCP) modules, a synonym for CUB and Sushi domains, is a common characteristic between five complement regulators (C4-binding protein, decay-accelerating factor, membrane co-factor protein, and complement receptors I and II). These proteins are known as regulators of complement activation (RCA), the largest group of complement regulators [52]. Inactivating C3b or dissociating C3 and C5 convertases form the regulatory mechanisms of RCA. The CCP module is the only identified domain in proteins that regulate the complement activity via C3 and C5 convertases. The rat orthologue of CSMD1 was identified as a novel multiple domain complement regulator through CCP sequence homologies [53]. The complement inhibitory capacity of CSMD1 was shown by a decreased amount of C3 on cell surfaces. CSMD1 only inhibited the classical and not the alternative pathway of the complement system. In situ hybridisation revealed *CSMD1* expression to be the highest in neurons in the CNS of adult rats [53]. 

A follow-up study investigated the complement inhibitory capacity of human CSMD1 [54]. Expression of a membrane-bound *CSMD1* fragment in Chinese hamster ovary (CHO) cells inhibited C3b and C9 deposition on the cell surface of CHO cells. Moreover, a soluble CSMD1 polypeptide interacted directly with C4b and C3b and significantly decreased C4b and C3b deposition in a concentration-dependent manner in human serum. The soluble construct also prevented C7 deposition on erythrocytes, through which CSMD1 potentially prevents MAC assembly [54]. Together, these data propose complement regulating proteins to potentially form risk factors in schizophrenia.

In conclusion, genetic associations with schizophrenia were found for RCA, which regulate the complement activity by dissociating C3 and C5 convertases. These findings further support the complement involvement in schizophrenia pathophysiology. 

## 8. Complement Protein C5

Reduced cortical grey matter thickness upon psychosis [26], and the identification of C4 as a schizophrenia-associated genetic variant [15], led to the assessment of complement gene mRNA expression correlation with cortical thickness in schizophrenia [55]. Increased *C5* and *Serpin Family G Member 1 (SERPING1)* expression were both found to be associated with reduced superior frontal thickness. The cleavage of C5 by C5 convertase produces C5a and C5b. C5a is an anaphylatoxin, just as C3a. Anaphylatoxins stimulate chemotaxis, and might recruit microglia in response to the reduced synaptic activity. *SERPING1* encodes the C1 inhibitor, thereby inhibiting complement activation. It is unexpected to find an inhibitor to be associated with the reduced cortical thickness, but this might represent a compensatory process [55]. 

The cerebral spinal fluid (CSF) of psychiatric patients was analysed to investigate whether C5 levels are affected in schizophrenia patients. CSF C5 levels were significantly increased in patients with a major depressive disorder and patients with schizophrenia compared to the healthy controls. CSF C5 levels were not significantly increased in patients with bipolar disorder compared to the controls. Increased CSF C5 could be the result of increased blood brain barrier permeability or increased C5 production in the CNS. However, some caution is warranted as CSF C5 levels also showed a positive correlation with antipsychotic medication dosage [56]. 

In another study, a protein analysis was conducted in blood samples taken from 12-year-old individuals who experienced psychotic events at the age of 18. Compared with age-matched controls, an upregulation of six proteins was seen in the patient group, comprising vitronectin, complement C1r subcomponent like protein, C8B, C8A, complement factor H, and C5 [57]. The increased C5 levels observed in individuals experiencing psychosis may reflect an increased complement activity during postnatal neurodevelopment. 

Schizophrenia and autoimmune disorders. If the complement system would indeed be crucial for proper brain maturation, the deficiency of one of its components should have a prominent impact on brain connectivity. Deficiency findings on several proteins belonging to the complement system reveal that the main diseases resulting from complement deficiencies are the autoimmune disorders systemic lupus erythematosus (SLE) and lupus-like disease [58]. In SLE, autoantibodies target antigens of nuclear components [59]. SLE is one of the four types of lupus. The three other types are neonatal, discoid, and drug-induced lupus. In lupus, immunological dysfunction causes multisystemic tissue inflammation [60]. Interestingly, a variant of SLE includes clinical manifestations as depression and psychosis. This variant is called neuropsychiatric lupus (NPSLE) [59]. Cognitive dysfunction is the most frequent psychiatric manifestation in SLE [61]. Although the pathogenesis of SLE is still being unraveled, current evidence points towards environmental factors triggering the innate and adaptive immune response in genetically susceptible individuals [62]. A loss of tolerance in both immune pathways causes immune responses directed at self-antigens, manifesting itself as multisystemic inflammation. 

There is reason to believe that inflammation also plays a role in schizophrenia when considering the signs of immune system activation [63], such as increased levels of pro-inflammatory markers such as cytokines [64,65]. Immune activity can be influenced by environmental factors, by stress or infections. Stress can activate microglia, which will evoke an immune response through pro-inflammatory cytokine secretion [66]. Infections increase the risk of schizophrenia [67,68]. Notably, autoimmune diseases are associated with an increased risk of schizophrenia [63,69]. This can be related to a shared genetic risk for schizophrenia and immune-mediated diseases [70]. Interestingly, it has also been suggested that inflammation affects dopaminergic neurotransmission [71], which would connect the dopamine and immunological/neuroimmune hypotheses. 

Taken together, susceptibility to schizophrenia might have an immunogenetic background [65]. Different theories about schizophrenia aetiology as increased synaptic pruning activity, environmental factors triggering disease onset, and hyperactivity of dopamine transmission could converge on the immune system dysregulation. However, a challenge in the association between immunity and schizophrenia is its directionality as immune disturbances can both be a cause and consequence of brain disruptions. 

## 9. Discussion

Strong evidence points towards a role for the complement system in postnatal development during the process of synapse elimination. The classical pathway of the complement system is not the only suggested pathway. Current evidence is convincing of a role for the complement system, through excessive pruning, in the observed cognitive deficits in schizophrenia. Involvement of the complement system would be in line with the hypothesis that disruption of the immune system contributes to the development of schizophrenia. 

**Figure 3:**
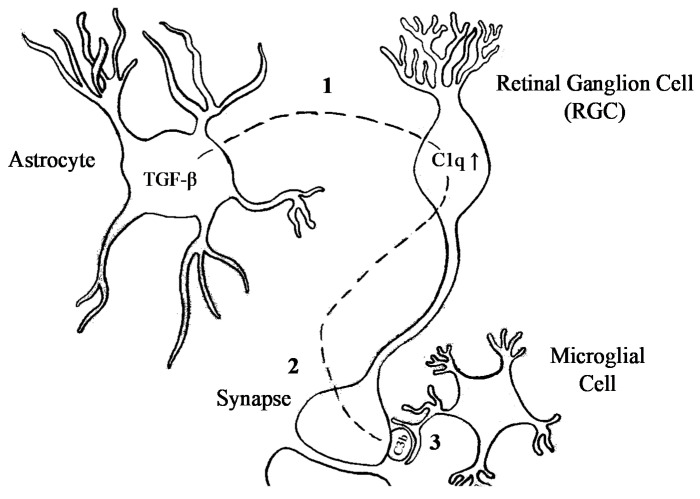
Schematic drawing of complement-mediated synapse elimination. (**1**) TGF-β excreted by astrocytes upregulates C1q expression in retinal ganglion cells (RGCs). (**2**) Increased C1q expression is followed by increased synaptic localisation of C3 (C3b). (**3**) C3 opsonised synapses are recognised by CR3s located on microglia.

C4 came forward as a contributing factor in schizophrenia via genetic studies, and subsequent functional studies suggested that increased levels of C4A and its effector C3 were associated with increased synaptic pruning in schizophrenia. The localisation of C1q and C3 at immature synapses [16] points towards a candidate mechanism involving an important role for astrocytes in synaptic refinement [16,72]. Exposure to astrocytes upregulated *C1q* expression by RGCs to tag cells destined for elimination. In the absence of C1q, RGC projections showed a segregation deficit, indicative of a lack of appropriate pruning in the dLGN. Since C3 deficiency provided the same phenotype as C1q, C1q-mediated pruning probably acts through the complement system [16]. Opsonisation of target cells triggers phagocytosing cells to engulf the opsonised cells. Microglia are the main phagocytosing cell of the CNS, which make them key candidates for synapse removal. Interactions between synapses and microglia in the dLGN are developmentally regulated and show activity-dependent microglial engulfment of RGC inputs by C3 [46]. The upstream regulation of the restricted expression of *C1q* during the pruning period is controlled by TGF-β secreted by astrocytes [34]. Taken together, these observations suggest that the secretion of TGF-β by astrocytes upregulates C1q in RGCs, leading to C3 opsonisation of synapses. These synapses are then recognised and engulfed by microglia (Figure 3). 

Apoptosis appears to be a major underlying molecular process through which synapses are eliminated by pruning [35]. A comparative proteomics analysis of C1q-tagged and untagged synapses showed that Annexin V competed with C1q for phosphatidylserine binding [35]. The C1q-phosphatidylserine binding suggests that C1q is not the initiating factor of synapse removal, but that it recognises synapses destined to die. Thus, apoptosis seems to be induced by an intrinsic mechanism, in response to a trigger that is yet to be defined. Considering the observation of activity-dependent synapse elimination, the reduced activity might very well be the upstream trigger of an intrinsic pathway in synapses that leads to apoptosis. 

Other pathways have been suggested to be responsible for pruning, as well. One alternative route consists of phagocytosis by astrocytes via multiple EGF-like-domains 10 (Megf10)/c-mer proto-oncogene tyrosine kinase (Mertk) pathways [73]. *MEGF10* and *MERTK*, which both have been linked to schizophrenia in association studies [74,75], encode phagocytic receptors trigger phagocytosis of target debris upon phosphatidylserine recognition [73]. Studies of the effect of apolipoprotein E (APOE) on astrocyte engulfment suggest that APOE regulates phagocytosis through APOE receptors on astrocytes. Since APOE receptors are located on microglia, it is possible that APOE is able to affect microglia-mediated phagocytosis [76]. However, some caution is warranted since these observations on defective pruning were made in the development of Alzheimer’s disease and it is not clear if APOE would have a similar involvement in synaptic pruning at earlier stages. Another potential pruning pathway would function through the fractalkine receptor [77]. In the CNS, the fractalkine receptor is only expressed by microglia [78]. Knocking out the fractalkine receptor resulted in delayed circuit maturation in the hippocampus of mice. The period of reduced synaptic pruning was accompanied by a significant reduction in microglia density [77], suggesting reduced microglial recruitment in the CNS. It is possible that the complement system and the fractalkine receptor act in parallel via microglia. Either C5a/C3a or fractalkine release can recruit microglia, which can, upon arrival, engulf and digest synapses targeted for degradation. 

Considering the multiple suggested pathways, a way to verify the importance of the complement system is to investigate the effect of its absence. In mouse models, knocking out proteins belonging to the classical pathway (C1q, C3, and C4) resulted in impaired segregation of ipsi- and contralateral projections from RGCs to the dLGN. However, looking at humans who are deficient for one of the complement proteins, only C1q deficiency seems to affect the CNS. What the deficiencies have in common is a moderate to high chance of developing SLE or lupus-like disease. SLE, in turn, increases the risk of developing schizophrenia [63]. More research is needed to elucidate the complex relation between schizophrenia and immune system disruptions. 

## 10. Conclusions

Strong evidence points towards a role for the complement system in postnatal development during the process of synapse elimination. Current evidence is convincing of a role for the complement system, through excessive pruning, in the observed cognitive deficits in schizophrenia. Before we can learn more about the contribution of complement activity, it must be determined whether complement activity is a response to phosphatidylserine exposure or that complement overactivity itself is a cause for excessive synaptic elimination. For that, additional complement protein knock-in and overexpression studies would be of great value for the assessment of the consequences of complement overactivity. In our view, two questions are of particular importance. First, is complement system overactivity a response to an increased amount of apoptotic synapses? If complement overactivity is only a response to an increased amount of apoptotic synapses, an intervention must be sought upstream of apoptosis initiation. GWASs indicate a genetically based overactivity of the complement system as a causative mechanism in a proportion of patients, but additional support will strengthen the primary role of the complement system. Second, is complement system hyperactivity itself causing excessive pruning? If complement system hyperactivity itself indeed causes excessive pruning, and if synapses are indeed pruned via microglia, a potential therapeutic intervention could be found in minocycline. If so, microglial engulfment and degradation of synapses could be inhibited by minocycline, which might be exploited for the development of therapeutic interventions for selective groups of schizophrenia patients.

## Data Availability

Not applicable.
